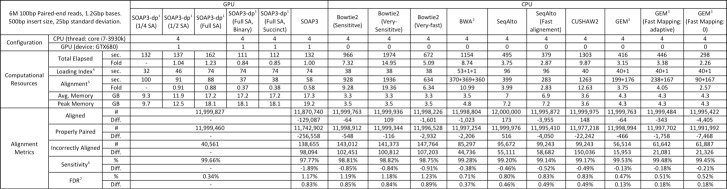# Correction: SOAP3-dp: Fast, Accurate and Sensitive GPU-Based Short Read Aligner

**DOI:** 10.1371/annotation/823f3670-ed17-41ec-ba51-b50281651915

**Published:** 2013-08-22

**Authors:** Ruibang Luo, Thomas Wong, Jianqiao Zhu, Chi-Man Liu, Xiaoqian Zhu, Edward Wu, Lap-Kei Lee, Haoxiang Lin, Wenjuan Zhu, David W. Cheung, Hing-Fung Ting, Siu-Ming Yiu, Shaoliang Peng, Chang Yu, Yingrui Li, Ruiqiang Li, Tak-Wah Lam

Table was presented incorrectly. A correct version of the Table is available here: 

**Figure:**