# Supplementary material for: SOAP3-dp: Fast, Accurate and Sensitive GPU-Based Short Read Aligner
Source: PLoS One. 2013 May 31;8(5):e65632. doi: 10.1371/journal.pone.0065632 (PMC3669295; doi:10.1371/journal.pone.0065632)
Supplement: File S1 — Supplementary Tables, Figures, Notes and Receipts. (DOC) [file pone.0065632.s003.doc]

*PLoS ONE*

**SOAP3-dp: Fast, accurate and sensitive GPU-based short read aligner**

Ruibang Luo, Thomas Wong, Jianqiao Zhu, Chi-Man Liu, Edward Wu, Haoxiang Lin, Lap-Kei Lee, Wenjuan Zhu, David W. Cheung, Hing-Fung Ting, Siu-Ming Yiu, Chang Yu, Yingrui Li, Ruiqiang Li, Tak-Wah Lam

| **Figure S1a,b** | | a) Cumulative recall rate; b) cumulative precision rate against mapping quality score from high to low of five aligners for simulated 100bp paired-end reads. |
| --- | --- | --- |
| **Figure S2** | | The comparison of the number of identified indels between BWA and SOAP3-dp (default parameters, PE100). |
| **Figure S3** | | Heat map of multi-nucleotide polymorphism calls enriched regions (SOAP3-dp against BWA). |
| **Figure S4** | | Multi-level alignment pipeline in SOAP3 module. |
| **Table S1** | | YH data production details. |
| **Table S2** | | Benchmark real data experiments: details of data characteristics and results of SOAP3-dp. |
| **Table S3** | | Benchmark real data experiments: results of BWA for paired-end reads and BWASW for single-end reads. |
| **Table S4** | | Benchmark real data experiments: results of Bowtie2. |
| **Table S5** | | Benchmark real data experiments: results of SeqAlto. |
| **Table S6** | | Benchmark real data experiments: results of GEM. |
| **Table S7** | | Benchmark real data experiments: results of BarraCUDA. |
| **Table S8** | | Benchmark real data experiments: results of CUSHAW. |
| **Table S9** | | Benchmark real data experiments: results of CUSHAW2. |
| **Table S10** | | Benchmark real data experiments: results of SOAP3. |
| **Table S11** | | Comparison on 14 sets of programs and parameters using 50bp paired-end simulated reads. |
| **Table S12** | | Comparison on 14 sets of programs and parameters using 75bp paired-end simulated reads. |
| **Table S13** | | Comparison on 14 sets of programs and parameters using 150bp paired-end simulated reads. |
| **Table S14** | | Comparison on 14 sets of programs and parameters using 250bp paired-end simulated reads. |
| **Table S15** | | Fosmid validation on 50 randomly selected SOAP3-dp specifically called deletions. |
| **Table S16** | | Fosmid validation details including Fosmid hits and alleles (in File Table S16.xls). |
| **Table S17** | | Number of MNP discovered by SOAP3-dp and BWA using two sets of parameters, with or without GATK’s local alignment. |
| **Supplementary Note** | | |
| **Command lines used to generate the tables (Receipts)** | | |
| **Data S1** | Assembled Fosmids sequences in BAM file (in File Data S1.bam). | |

*Note: Software is available at http://www.cs.hku.hk/2bwt-tools/soap3-dp/*

# Supplementary Figures

a.


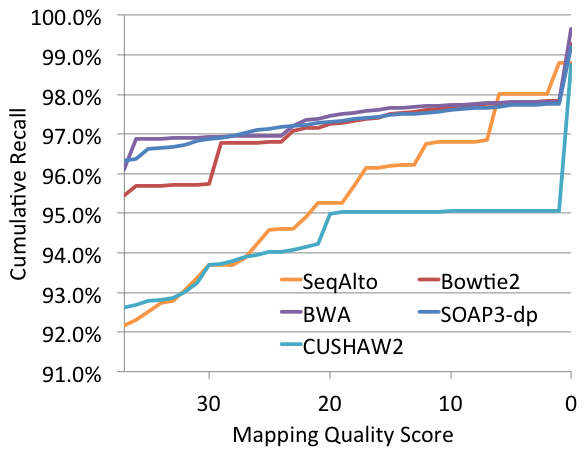


b.


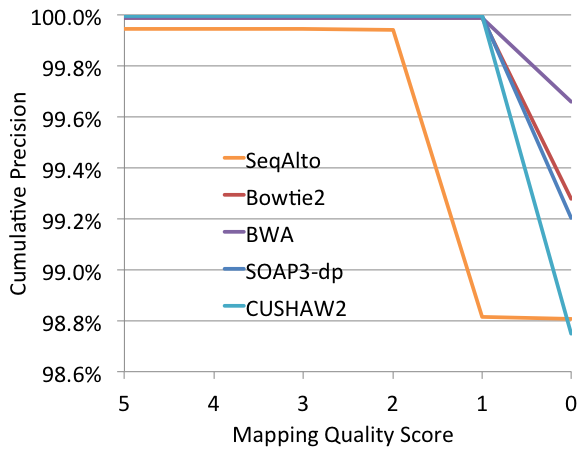


Figure S1, **a**) cumulative recall rate (# of reads correctly aligned / total # of reads); **b**) cumulative precision rate (# of reads correctly aligned / # of reads aligned) against mapping quality score from high to low of five aligners including Bowtie2, BWA, SeqAlto, SOAP3-dp and CUSHAW2 for simulated 100bp paired-end reads.


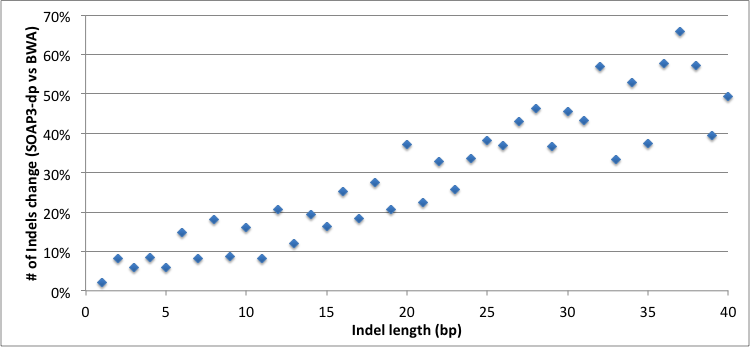


Figure S2, The comparison of the number of identified indels between BWA and SOAP3-dp (default parameters, PE100). Y-axis is calculated by $\frac{SOAP3dp-BWA}{SOAP3dp+BWA}$.


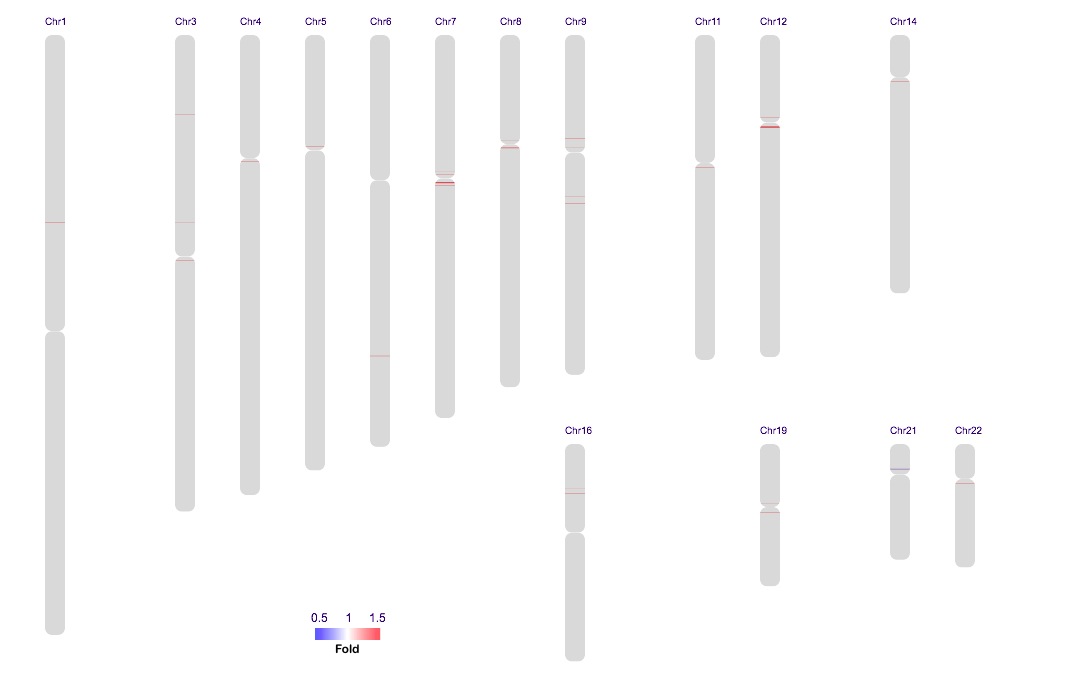


Figure S3, Heat map showing genome regions with additional multi-nucleotide polymorphisms (MNP) identified specifically by using SOAP3-dp alignment. The intensity of red shows how many folds of MNP did SOAP3-dp detected more than BWA in 100kbp-sliding windows.


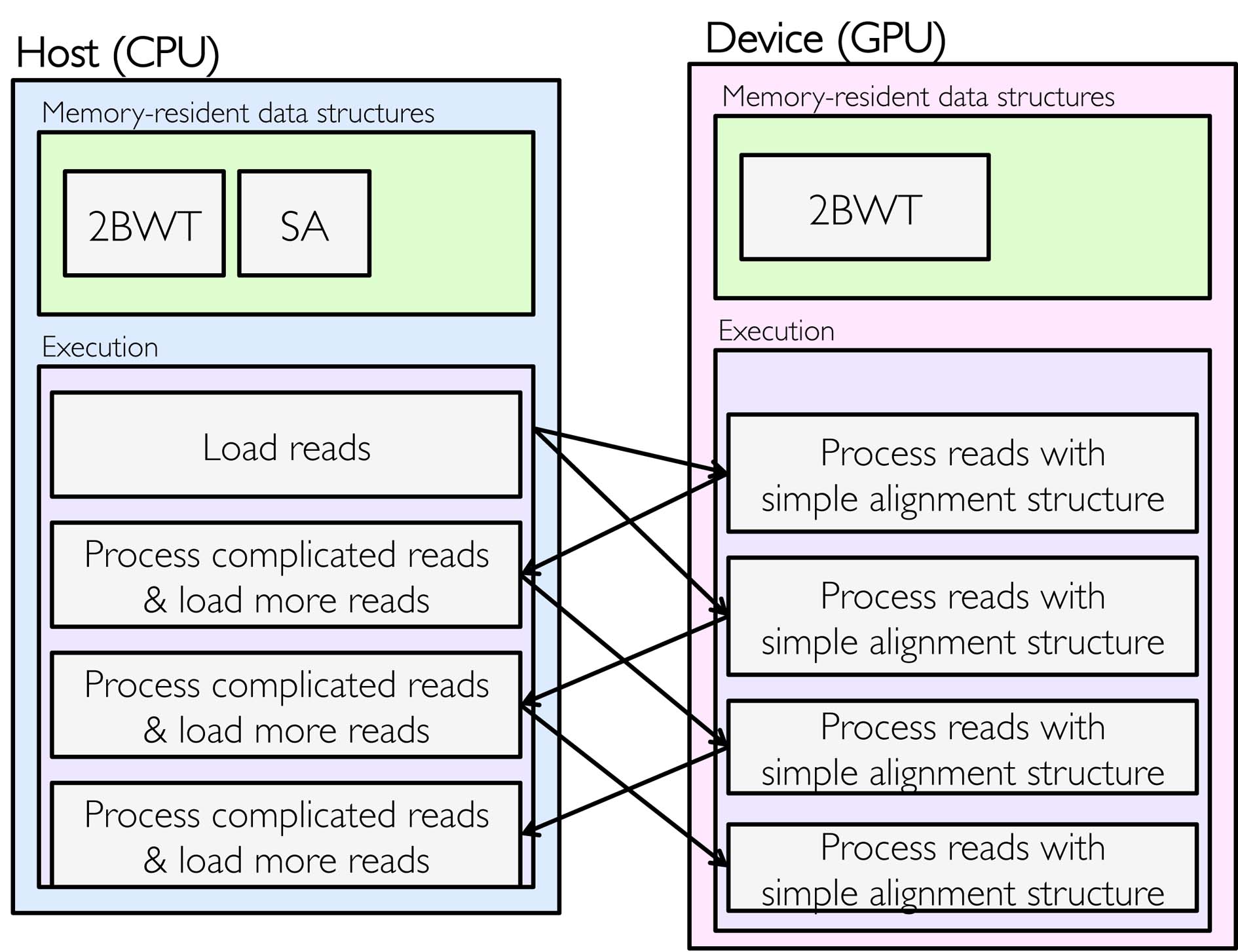


Figure S4, Multi-level alignment pipeline in SOAP3[^1^](#_ENREF_1) module.

A parameter is devised to determine at runtime whether a read would generate too many branches. Different thresholds on the parameter are used to differentiate the complexity of the reads. We stop the execution of the complicated reads in GPU, group them and redo the alignment of them in CPU to decrease the amount of idle time of the processors in GPU. Moreover, SOAP3 overlaps the alignment of complicated reads from the previous batch in CPU with the alignment of the next batch in GPU, in order to keep both GPU and CPU busy all the time.

# Supplementary Tables

Table S1, YH data production details.


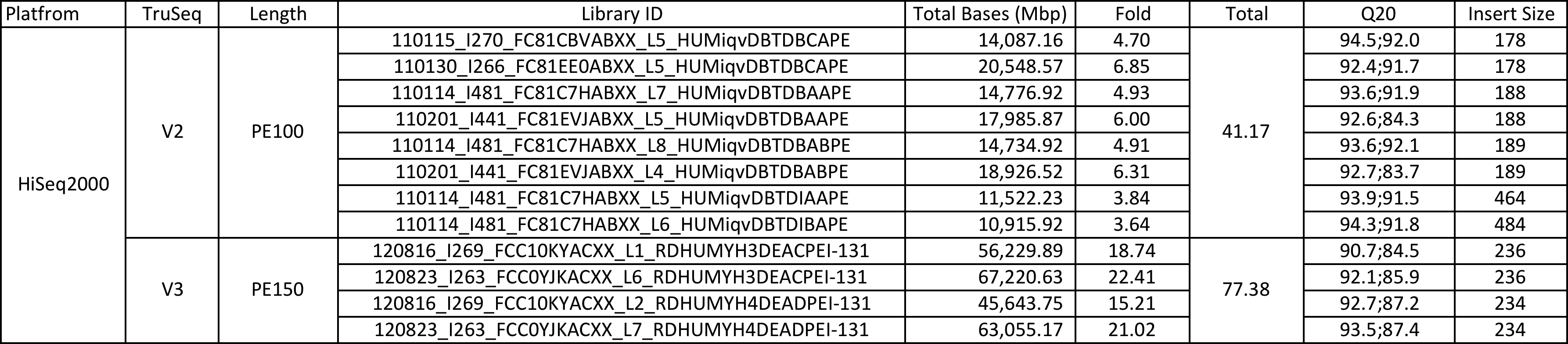


Table S2, Benchmark real data experiments: details of data characteristics and the results of SOAP3-dp.


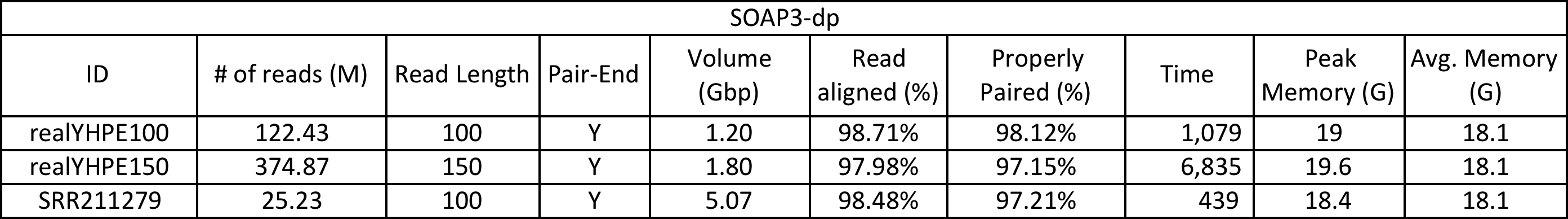


Table S3, Benchmark real data experiments: results of BWA for paired-end reads. The “CMP” columns after “Read aligned”, “Properly paired” and “Total time” show the difference between BWA and SOAP3-dp.

Table S4, Benchmark real data experiments: results of Bowtie2. The “CMP” columns after “Read aligned”, “Properly paired” and “Total time” show the difference between Bowtie2 and SOAP3-dp.


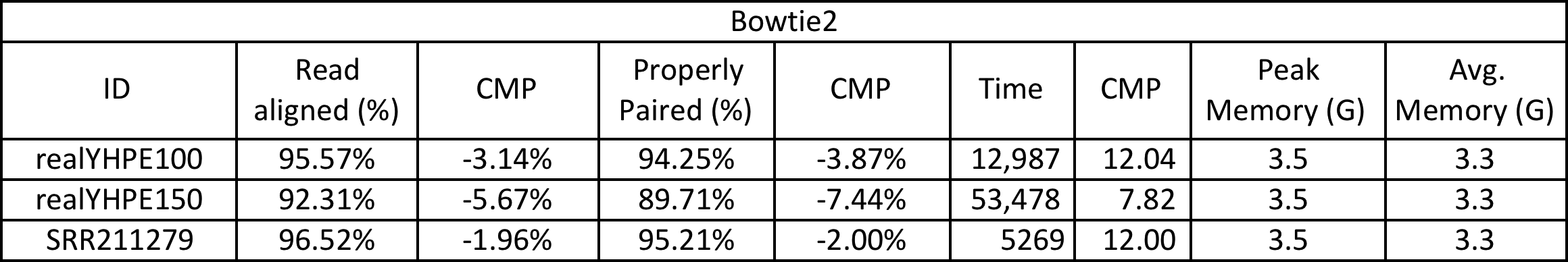


Table S5, Benchmark real data experiments: results of SeqAlto. The “CMP” columns after “Read aligned”, “Properly paired” and “Total time” show the difference between SeqAlto and SOAP3-dp.


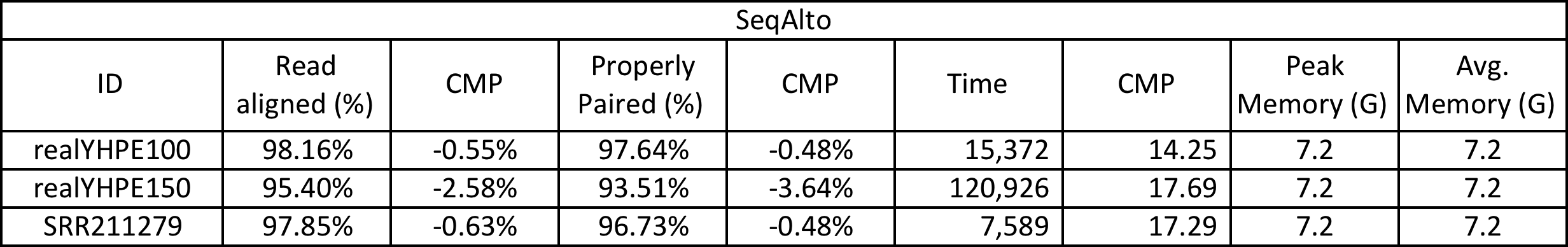


Table S6, Benchmark real data experiments: results of GEM. The “CMP” columns after “Read aligned”, “Properly paired” and “Total time” show the difference between GEM and SOAP3-dp.


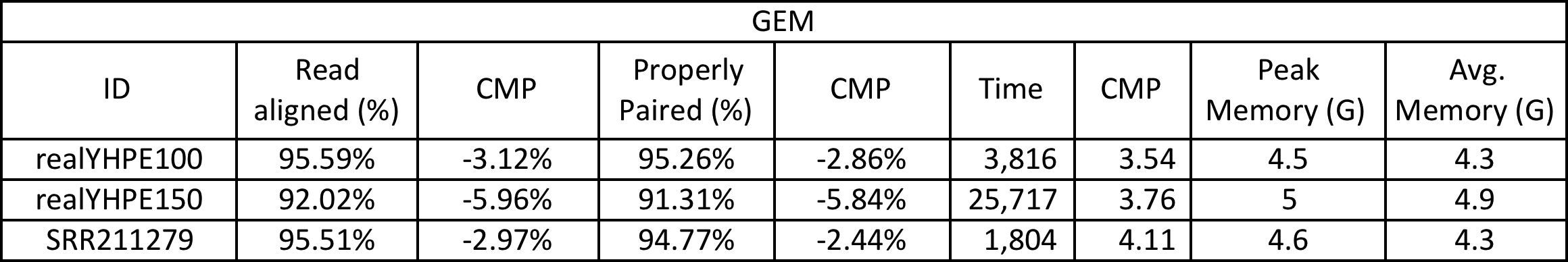


Table S7, Benchmark real data experiments: results of BarraCUDA. The “CMP” columns after “Read aligned”, “Properly paired” and “Total time” show the difference between BarraCUDA and SOAP3-dp.


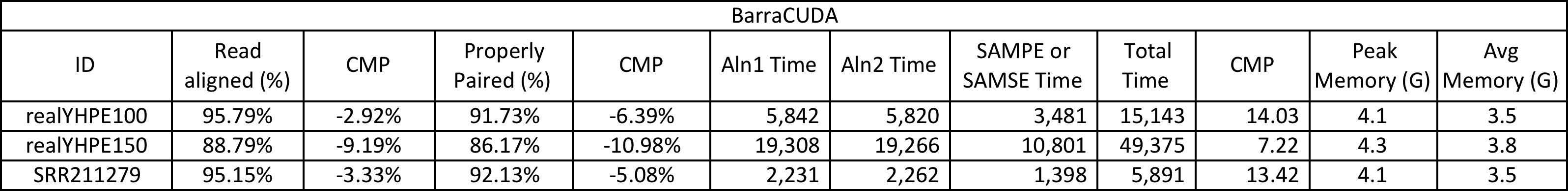


Table S8, Benchmark real data experiments: results of CUSHAW. The “CMP” columns after “Read aligned”, “Properly paired” and “Total time” show the difference between CUSHAW and SOAP3-dp.


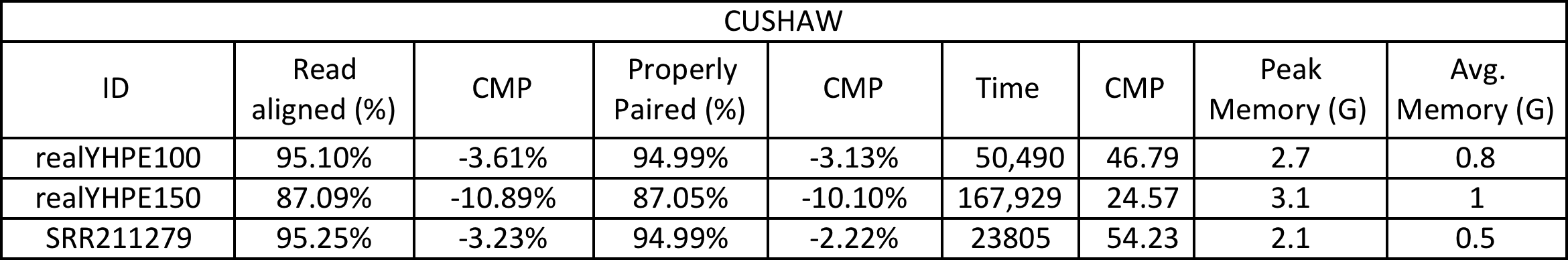


Table S9, Benchmark real data experiments: results of CUSHAW2. The “CMP” columns after “Read aligned”, “Properly paired” and “Total time” show the difference between CUSHAW2 and SOAP3-dp.


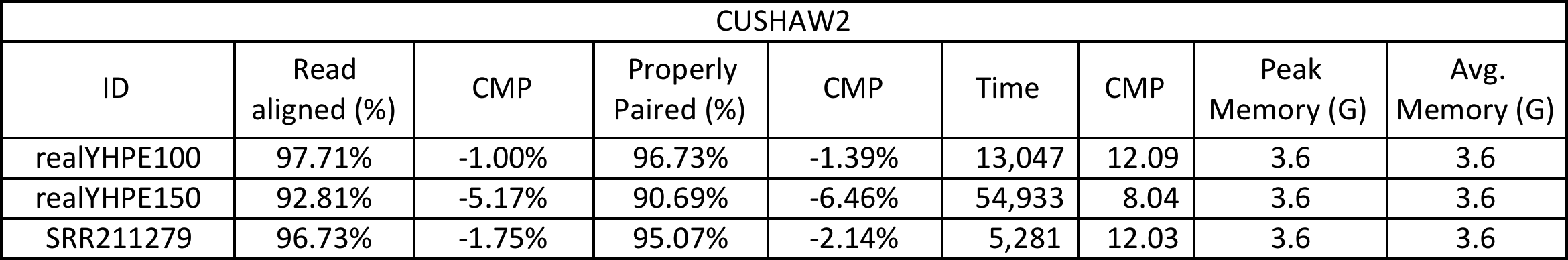


Table S10, Benchmark real data experiments: results of SOAP3. The “CMP” columns after “Read aligned”, “Properly paired” and “Total time” show the difference between SOAP3 and SOAP3-dp.


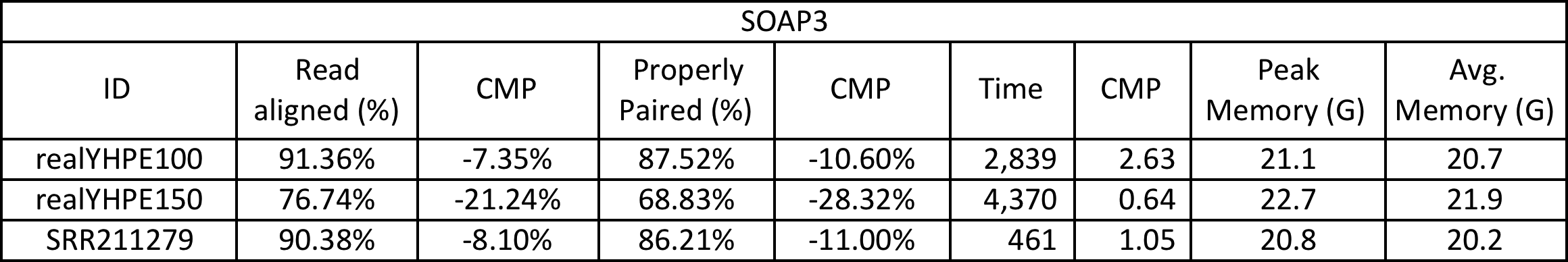


Table S11, Comparison on 14 sets of programs and parameters using 50bp paired-end simulated reads.


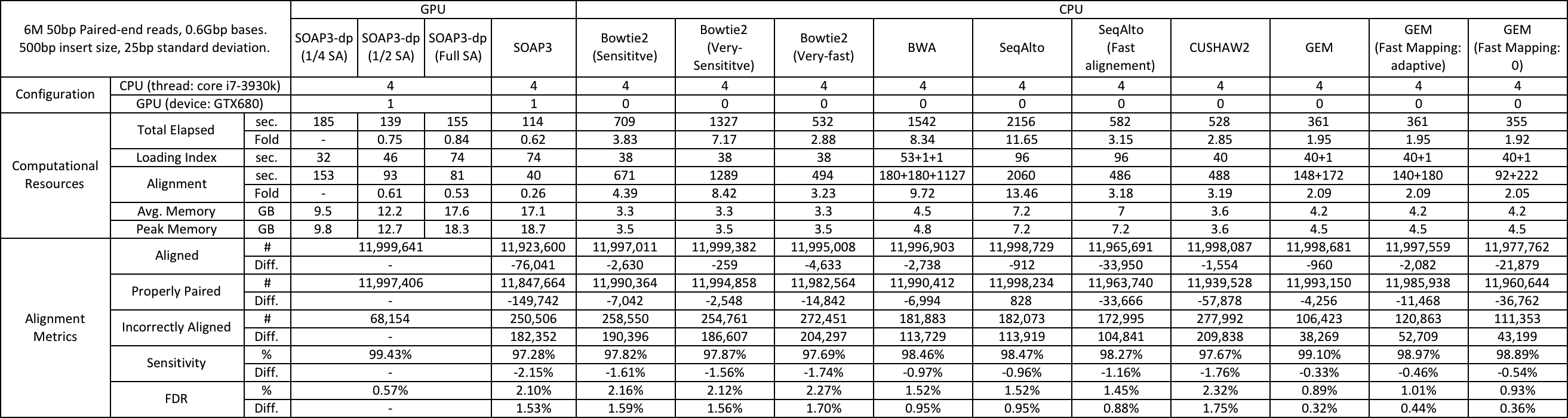


Table S12, Comparison on 14 sets of programs and parameters using 75bp paired-end simulated reads.


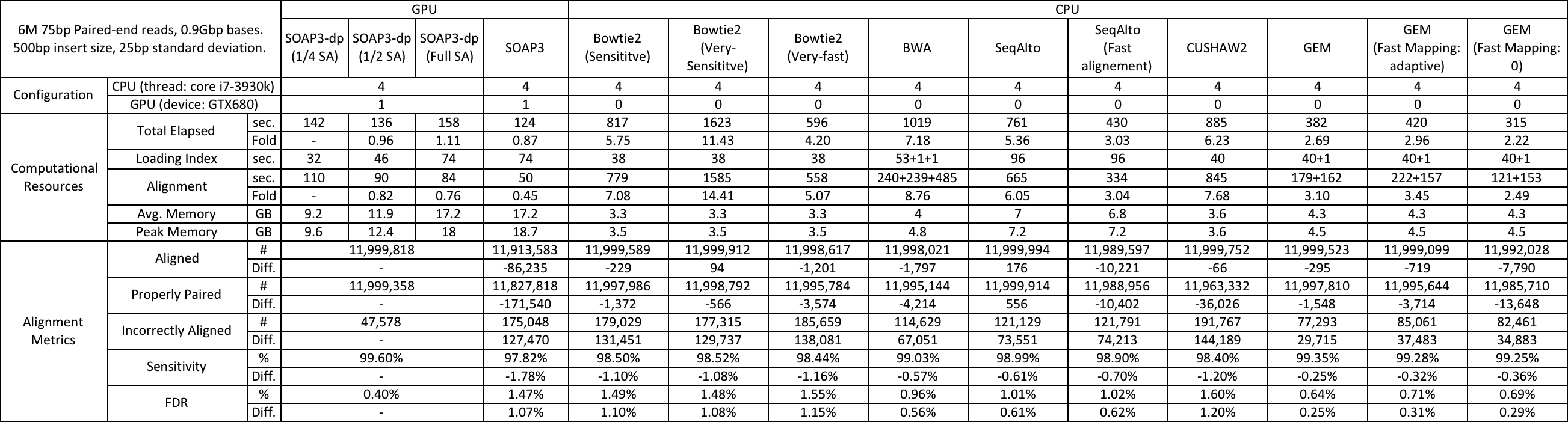


Table S13, Comparison on 14 sets of programs and parameters using 150bp paired-end simulated reads.


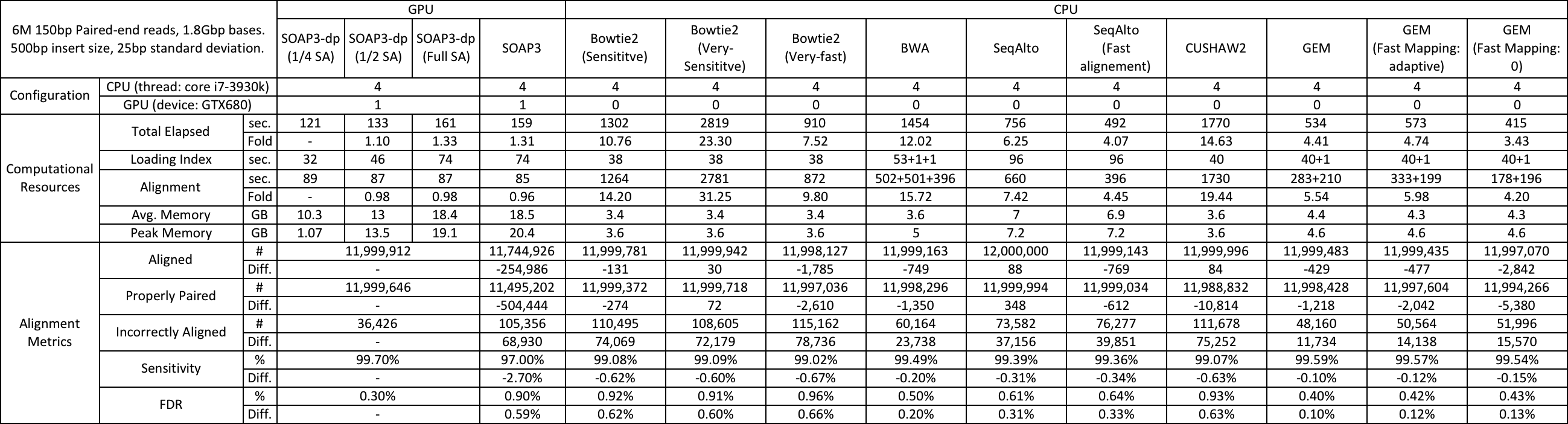


Table S14, Comparison on 14 sets of programs and parameters using 250bp paired-end simulated reads.


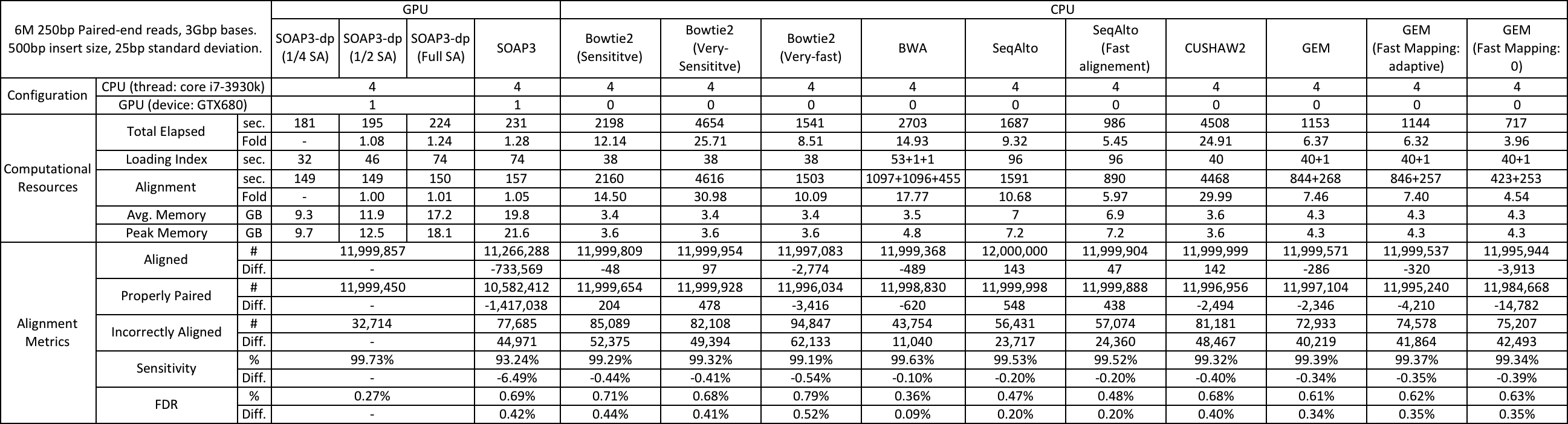


Table S15, Fosmid validation on 50 randomly selected SOAP3-dp specifically called deletions.


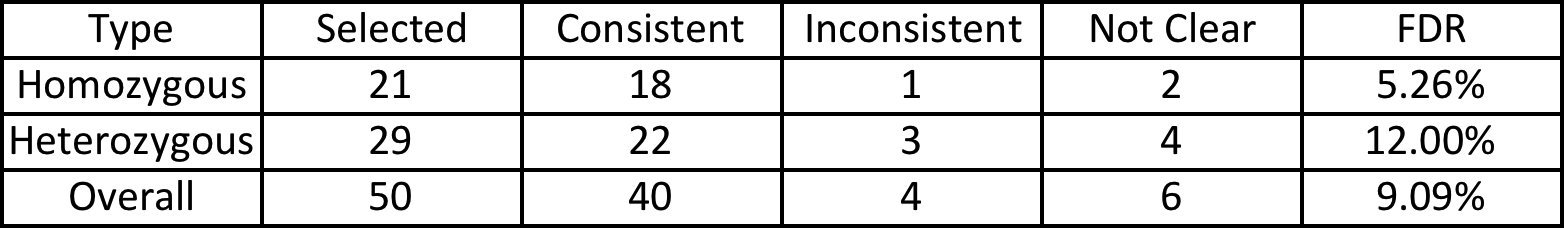


Table S16, Fosmid validation details including Fosmid hits and alleles.

<Table S16 in File Table S16.xls>

Table S17, Number of MNP discovered by SOAP3-dp and BWA using two sets of parameters, with or without GATK’s local alignment.


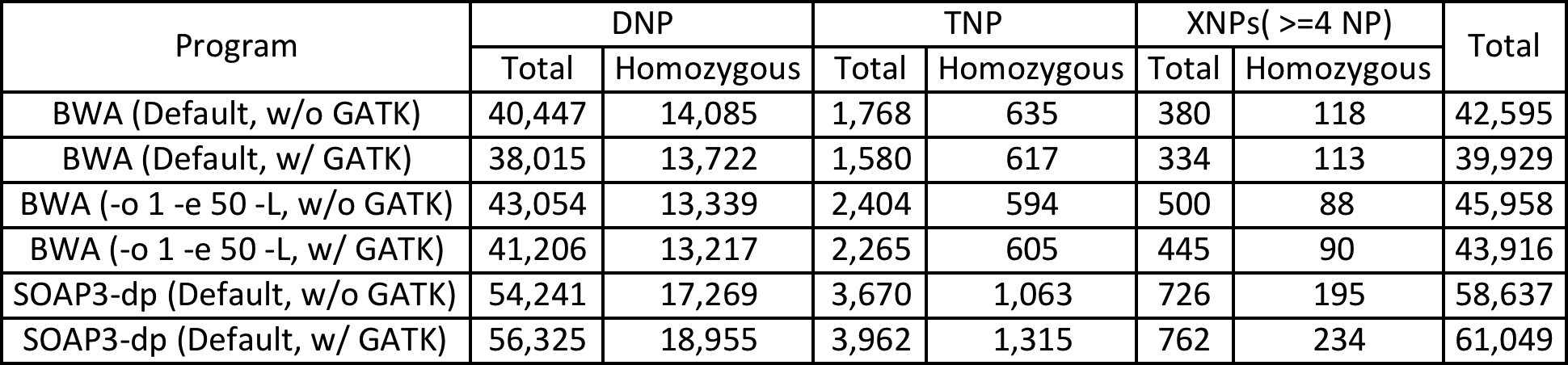


# Supplementary Note

1. Scoring function.

SOAP3-dp provides two sets of scoring function.

1) MAQ and BWA compliant scoring function (Default).

2) Strict scoring function reports the mapping quality score of an alignment in a range from 0 to 40 (could be adjusted by parameters). The higher the score, the more reliable the alignment is. An alignment is given a score only if it is the best alignment. For a single alignment, the score is mainly determined by the uniqueness and the mapped quality of the alignment. High level of uniqueness requires: (1) the best alignment is unique; (2) there does not exist any other alignment with DP score close to that of the best alignment; (3) only limited number of other alignments are reported and their DP scores are relatively low. On the other hand, good mapping quality of the alignment is based on high DP score and low base quality values on the mismatch positions. For a paired-end alignment, the score depends on the uniqueness and the mapped quality of the paired-end alignment as well as the single alignment of each end. In general, the computation of the mapping quality score considers all these factors, and the best alignment, either single or paired-end, mapped in good quality with high level of uniqueness will be awarded a high score.

1. Chimeric read alignment

SOAP3-dp performs global alignment thus does not detect chimeric alignments, though the method can be extended to support this; for example, reads aligned with over half of the read soft-clipped could be saved and then matched with other sub-optimal alignments for chimeric alignments. Support for chimeric alignment is future work.

1. Running SOAP3-dp on Amazon EC2

Amazon elastic compute cloud (EC2) provides various machine instance types and storage approaches, which makes it flexible enough to carry out different scales of computation. Nowadays, Amazon EC2 hosts plenty of bioinformatics researches and applications. Notably, 200 terabytes of data generated by 1000 genome project was achieved natively in EC2, which substantially expedited future genome studies. Recently, EC2 started to provide “GPU instance”, which costs $2.1 dollars per hour. Each instance provides 8 physical CPU cores, 2 NVIDIA Tesla M2050, 22 gigabytes of memory, 1.7 terabyte local storage and 10Gbps Ethernet connection to network and external storages.

To test SOAP3-dp’s performance on Amazon EC2, we’ve selected 10 datasets (ERR126299, ERR126300, SRR211276, SRR211272, ERR125594, ERR125595, ERR068424, ERR068422, SRR493233, ERR068421) from 1000 genome projects. These paired-end reads were sequenced using Illumina HiSeq 2000 by different institutes with 100bp read length. The total volume is 131.44Gbp (43.8-fold human genome). The alignment of the 10 datasets was distributed to the two Tesla M2050 cards in balance by sharing the same copy of index in host memory. Each run of soap3-dp uses at most 7 cores of CPU. SOAP3-dp uses locked memory when sharing index in host memory, which is not swappable and limited by operating system by default. Security policy should be changed by 1) running command “ulimit –l unlimited” or 2) simply adding a row “* - memlock unlimited” to the system resource configuration file and then re-login using a new terminal (distribution dependent, “/etc/security/limits.conf” for Amazon AMI for example).

Notably, soap3-dp uses asynchronous read but synchronous write. This is especially suitable for Amazon EC2, where the massive amount of raw data (e.g. 1000 genome project data) was usually on-line retrieved from the relatively slower Amazon simple storage service (S3) or EBS (Elastic Block Storage), and the results could be written to RAID-enabled local storage. In our test, we read the raw reads from Amazon elastic block storage (EBS), which is slower, and write the results to local RAID-0 storage.

SOAP3-dp ran using default parameters and BAM output. The alignment finished in 3.8 hours, with only a couple of seconds waiting for I/O. Yielding a total cost of $7.98, or $0.061 per Gbp reads aligned.

# Command lines used to generate the tables (Receipts)

Caches were cleared before every invocation of the programs using:

sysctl -w vm.drop_caches=3

**Benchmark for SOAP3-dp (v2.3)**

Building Index with full SA, ½ SA or ¼ SA using “soap3-dp-builder (Step 1) and BGS-Build (Step 2)”:

Full SA: Modify “SaValueFreq” to “1” in configuration file “soap3-dp-builder.ini” before running soap3-dp-builder.

½ SA: Modify “SaValueFreq” to “2” in configuration file “soap3-dp-builder.ini” before running soap3-dp-builder.

¼ SA: Modify “SaValueFreq” to “4” in configuration file “soap3-dp-builder.ini” before running soap3-dp-builder.

Simulated reads alignment:

Command line: “soap3-dp pair genome.fa.index _1.fq _2.fq -u 650 -v 350 -o *soap3dp* -L *$max_read_length*”

Configuration file: “NumOfCpuThreads=4, BWALikeScore=1, ShareIndex=1, MaxFrontLenClipped=49, MaxEndLenClipped=49”

Real reads alignment:

Command line: “soap3-dp pair genome.fa.index _1.fq _2.fq -u 1000 -v 1 -o *soap3dp* -L *$max_read_length*”

Configuration file: “NumOfCpuThreads=4, BWALikeScore=1, ShareIndex=1, MaxFrontLenClipped=49, MaxEndLenClipped=49”

**Benchmark for BWA (v0.6.2)**

Simulated reads with default parameters:

bwa aln -t 4 genome.fa _1.fq >_1.aln

bwa aln -t 4 genome.fa _2.fq >_2.aln

bwa sampe genome.fa _1.aln _2.aln _1.fq _2.fq >*bwa.sam*

Real reads with default parameters:

bwa aln -t 4 –I genome.fa _1.fq >_1.aln

bwa aln -t 4 –I genome.fa _2.fq >_2.aln

bwa sampe genome.fa _1.aln _2.aln _1.fq _2.fq >*bwa.sam*

Real reads with parameters to allow “a gap no longer than 50bp”:

bwa aln -t 4 –I –o 1 –e 50 –L genome.fa _1.fq >_1.aln

bwa aln -t 4 –I –o 1 –e 50 –L genome.fa _2.fq >_2.aln

bwa sampe genome.fa _1.aln _2.aln _1.fq _2.fq >*bwa.sam*

**Benchmark for Bowtie2 (v2.0.0-beta4)**

Simulated reads:

Sensitive:

bowtie2 -x main.fa -1 _1.fq -2 _2.fq --sensitive -S *bowtie2-sensitive* -I 350 -X 650 -p 4

Very sensitive:

bowtie2 -x main.fa -1 _1.fq -2 _2.fq --very-sensitive -S *bowtie2-very-sensitive* -I 350 -X 650 -p 4

Very fast:

bowtie2 -x main.fa -1 _1.fq -2 _2.fq --very-fast -S *bowtie2-very-fast* -I 350 -X 650 -p 4

Real reads:

bowtie2 -x main.fa -1 _1.fq -2 _2.fq -S *bowtie2* -I 1 -X 1000 -p 4

Details for critical parameters (copied from the usage of Bowtie2):

--very-fast -D 5 -R 1 -N 0 -L 22 -i S,0,2.50

--sensitive -D 15 -R 2 -N 0 -L 22 -i S,1,1.15

--very-sensitive -D 20 -R 3 -N 0 -L 20 -i S,1,0.50

-N <int> max # mismatches in seed alignment; can be 0 or 1 (0)

-L <int> length of seed substrings; must be >3, <32 (22)

-i <func> interval between seed substrings w/r/t read len

(S,1,1.15)

-D <int> give up extending after <int> failed extends in a row

(15)

-R <int> for reads w/ repetitive seeds, try <int> sets of

seeds (2)

**Benchmark for SeqAlto (basic 0.5-r123)**

Simulated reads:

Default:

seqalto_basic align genome.fa.midx -1 _1.fq -2 _2.fq -p 4 -m 500 -i 650 >*seqalto.sam*

Fast alignment:

seqalto_basic align genome.fa.midx -1 _1.fq -2 _2.fq -p 4 -m 500 -i 650 >*seqalto.sam*

Real reads:

seqalto_basic align genome.fa.midx -1 _1.fq -2 _2.fq -p 4 -m 500 -i 1000 >*seqalto.sam*

Details for critical parameters (copied from the usage of SeqAlto):

-f -- Fast alignment

**Benchmark for BarraCUDA (r232, r260)**

Simulated reads and Real reads:

barracuda aln main.fa _1.fq >_1.aln

barracuda aln main.fa _2.fq >_2.

barracuda sampe main.fa b_1.aln b_2.aln _1.fq _2.fq >*barracuda*

**Benchmark for CUSHAW (v1.0.40)**

Simulated reads:

cushaw-long *cushaw* main.fa -fastqPaired _1.fq _2.fq -all_in_sam -i 650 -t 4

Real reads:

cushaw-long *cushaw* main.fa -fastqPaired _1.fq _2.fq -all_in_sam -i 1000 -t 4

**Benchmark for CUSHAW (v2.1.9)**

Simulated reads and real reads:

cushaw2 -r main.fa -q _1.fq _2.fq -o *cushaw2* -t 4

**Benchmark for SOAP3 (version146)**

Simulated reads:

Command line: “soap3_aligner pair genome.fa.index _1.fq _2.fq -u 650 -v 350 -o *soap3* –b 2 -L *$max_read_length*”

Configuration file: “NumOfCpuThreads=4”

Real reads:

Command line: “soap3_aligner pair genome.fa.index _1.fq _2.fq -u 1000 -v 1 -o *soap3* –b 2 -L *$max_read_length*”

Configuration file: “NumOfCpuThreads=4”

**Benchmark for GEM (core_i3-20121106-022124)**

Simulated reads:

Default:

gem-mapper -I main.gem -1 _1.fq -2 _2.fq -o gem -q offset-33 -p --min-insert-size 350 --max-insert-size 650 -T 4 -m 0.04 -e 0.04 -s 0 -p -E 0.30

gem-map-2-map -I main -i gem.map -s -b,-h,-a,-s | gem-2-sam -I main -q offset-33 -o *gem.sam* -c --threads 4

Adaptive fast mapping:

gem-mapper -I main.gem -1 _1.fq -2 _2.fq -o *gem* -q offset-33 -p --min-insert-size 350 --max-insert-size 650 -T 4 -m 0.04 -e 0.04 -s 0 -p -E 0.30 --fast-mapping

gem-map-2-map -I main -i gem.map -s -b,-h,-a,-s | gem-2-sam -I main -q offset-33 -o *gem.sam* -c --threads 4

Fastest mapping:

gem-mapper -I main.gem -1 _1.fq -2 _2.fq -o *gem* -q offset-33 -p --min-insert-size 350 --max-insert-size 650 -T 4 -m 0.04 -e 0.04 -s 0 -p -E 0.30 --fast-mapping=0

gem-map-2-map -I main -i gem.map -s -b,-h,-a,-s | gem-2-sam -I main -q offset-33 -o *gem.sam* -c --threads 4

100bp simulated reads using SOAP3-dp’s default parameters:

Default:

gem-mapper -I main.gem -1 _1.fq -2 _2.fq -o gem -q offset-33 -p --min-insert-size 350 --max-insert-size 650 -T 4 -m 22 -e 0.40 -s 0 -p -E 0.40

gem-map-2-map -I main -i gem.map -s -b,-h,-a,-s | gem-2-sam -I main -q offset-33 -o *gem.sam* -c --threads 4

Adaptive fast mapping:

gem-mapper -I main.gem -1 _1.fq -2 _2.fq -o gem -q offset-33 -p --min-insert-size 350 --max-insert-size 650 -T 4 -m 22 -e 0.40 -s 0 -p -E 0.40 --fast-mapping

gem-map-2-map -I main -i gem.map -s -b,-h,-a,-s | gem-2-sam -I main -q offset-33 -o *gem.sam* -c --threads 4

Fastest mapping:

gem-mapper -I main.gem -1 _1.fq -2 _2.fq -o gem -q offset-33 -p --min-insert-size 350 --max-insert-size 650 -T 4 -m 22 -e 0.40 -s 0 -p -E 0.40 --fast-mapping=0

gem-map-2-map -I main -i gem.map -s -b,-h,-a,-s | gem-2-sam -I main -q offset-33 -o *gem.sam* -c --threads 4

Real reads:

gem-mapper -I main.gem -1 _1.fq -2 _2.fq -o gem -q offset-64 -p -T 4 -m 0.04 -e 0.04 -s 0 -p -E 0.30

gem-map-2-map -I main -i gem.map -s -b,-h,-a,-s | gem-2-sam -I main -q offset-64 -o *gem.sam* -c --threads 4

SRR211279 reads:

gem-mapper -I main.gem -1 _1.fq -2 _2.fq -o gem -q offset-33 -p -T 4 -m 0.04 -e 0.04 -s 0 -p -E 0.30

gem-map-2-map -I main -i gem.map -s -b,-h,-a,-s | gem-2-sam -I main -q offset-33 -o *gem.sam* -c --threads 4

Details for critical parameters (copied from the usage of GEM):

--fast-mapping <number>|'adaptive' (default=false)
